# Supplementary material for: Differential Expression of microRNAs in Serum of Patients with Chronic Painful Polyneuropathy and Healthy Age-Matched Controls
Source: Biomedicines. 2023 Mar 2;11(3):764. doi: 10.3390/biomedicines11030764 (PMC10045018; doi:10.3390/biomedicines11030764)
Supplement: Supplementary file 1 [file biomedicines-11-00764-s001.zip › biomedicines-2143919-supplementary.pdf]

Table S1. Logistic regression, Tabular Results, Prism 9.4.1 (GraphPad, Boston, MA, USA).

	***hsa*-miR-3135b**	***hsa*-miR-584-5p**	***hsa*-miR-12136**	***hsa*-miR-550a-3p**
**Best-fit values**				
β0	3.21	1.032	3.719	-2.326
β1	-0.1261	-0.001814	-0.3208	0.2813
X at 50%	25.45	569.1	11.59	8.268
**Std. Error**				
β0	0.7925	0.4504	0.9899	0.8101
β1	0.03222	0.0007172	0.08746	0.09686
X at 50%	3.298	158.7	1.096	1.028
**95% CI (profile likelihood)**				
β0	1.824 to 4.991	0.1943 to 1.973	1.975 to 5.911	-4.051 to -0.8492
β1	-0.2001 to -0.07152	-0.003422 to -0.0006064	-0.5168 to -0.1696	0.1114 to 0.4945
X at 50%	19.48 to 32.94	203.3 to 1024	9.515 to 14.16	5.989 to 10.88
**Odds ratios**				
β0	24.77	2.807	41.21	0.0977
β1	0.8815	0.9982	0.7256	1.325
**95% CI (profile likelihood) for odds ratios**				
β0	6.198 to 147.0	1.214 to 7.191	7.209 to 369.0	0.01740 to 0.4278
β1	0.8186 to 0.9310	0.9966 to 0.9994	0.5964 to 0.8440	1.118 to 1.640
**Is slope significantly non-zero?**				
|Z|	3.914	2.529	3.668	2.905
P value	<0.0001	0.0115	0.0002	0.0037
Deviation from zero?	Significant	Significant	Significant	Significant
**Likelihood ratio test**				
Log-likelihood ratio (G squared)	41.54	11.04	30.47	12.18
P value	<0.0001	0.0009	<0.0001	0.0005
Reject Null Hypothesis?	Yes	Yes	Yes	Yes
P value summary	****	***	****	***
**Area under the ROC curve**				
Area	0.9139	0.6956	0.8783	0.785
Std. Error	0.04125	0.06923	0.0477	0.06051
95% confidence interval	0.8330 to 0.9947	0.5599 to 0.8312	0.7848 to 0.9718	0.6664 to 0.9036
P value	<0.0001	0.0093	<0.0001	0.0001
**Goodness of Fit**				

Tjur's R squared	0.5763	0.1589	0.4386	0.2067
Cox-Snell's R squared	0.4996	0.1681	0.3982	0.1837
Model deviance, G squared	41.63	72.13	52.71	71
**Equation**	log odds = 3.210-0.1261*X	log odds = 1.032-0.001814*X	log odds = 3.719-0.3208*X	log odds = -2.326+0.2813*X
**Data summary**				
Rows in table	60	60	60	60
Rows skipped (missing data)	0	0	0	0
Rows analyzed (#observations)	60	60	60	60
Number of 1	30	30	30	30
Number of 0	30	30	30	30
Number of parameter estimates	2	2	2	2
#observations/#parameters	30	30	30	30
# of 1/#parameters	15	15	15	15
# of 0/#parameters	15	15	15	15
